# The regulatory role of exercise in heart failure and myocardial energy metabolism: A review

**DOI:** 10.17305/bb.2025.12072

**Published:** 2025-02-24

**Authors:** Yuanhao Li, Dongli Gao, Peixia Li, Xulei Duan, Youli Liu, Chengyan Wu, Libo Wang, Xuehui Wang

**Affiliations:** 1 The First Affiliated Hospital of Xinxiang Medical University, Department of Cardiology, Xinxiang, Henan, China; 2 The Third Affiliated Hospital of Xinxiang Medical University, Department of Cardiology, Xinxiang, Henan, China

**Keywords:** Exercise, heart failure, HF, metabolism, mitochondria, cardiac remodeling, cardiac rehabilitation

## Abstract

Myocardial energy metabolism is crucial for maintaining optimal heart function. The heart, having limited energy storage capacity, is dependent on a continuous energy supply; any disruptions or alterations in energy metabolism pathways can lead to insufficient myocardial energy, potentially triggering heart failure (HF). Exercise, as a safe and economical non-pharmacological intervention, is widely recognized to enhance cardiovascular health and modify myocardial energy metabolism patterns. However, the specific mechanisms by which exercise regulates myocardial metabolism to prevent and treat HF remain unclear. This review aims to detail the characteristics of myocardial metabolism under normal physiological and HF conditions, to further explore the impact of different exercise modalities on myocardial metabolism, and to summarize the molecular mechanisms by which exercise protects the heart by optimizing myocardial energy metabolism. Ultimately, this article aims to provide an in-depth understanding and evidence for the application of exercise interventions in cardiac rehabilitation.

## Introduction

The heart, which beats billions of times over a lifetime and consumes substantial amounts of adenosine triphosphate (ATP) daily, depends on flexible metabolic pathways due to its limited immediate energy reserves. This metabolic adaptability is crucial for maintaining cardiac function [1]. As a high-energy-demanding organ, any disruption or alteration in myocardial energy metabolism can have severe consequences on heart function. Consequently, an imbalance in energy metabolism is recognized as a key factor in the progression of heart failure (HF) [2]. Exercise, widely regarded as a safe and cost-effective non-pharmacological intervention, offers significant cardiovascular benefits by promoting physiological ventricular remodeling and influencing myocardial energy metabolism. While exercise is known to affect myocardial metabolism, its precise protective role against HF remains unclear, likely due to the complexity of myocardial metabolic networks [3].

**Figure 1.**
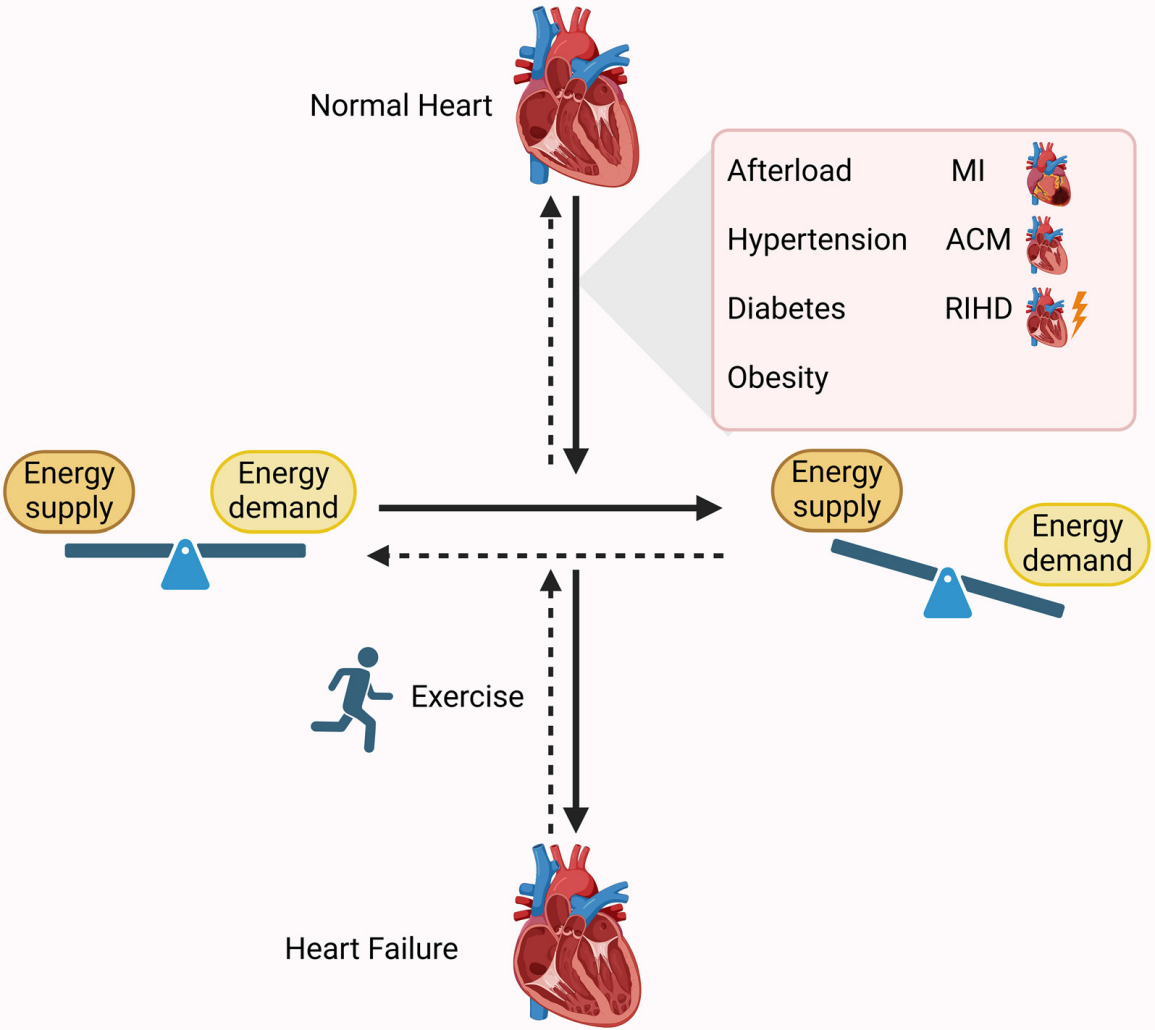
**Exercise regulates myocardial metabolic imbalance to improve cardiac function.** Pathological factors, such as hypertension, increased afterload, diabetes, obesity, MI, ACM, and RIHD, can lead to an imbalance in myocardial energy metabolism supply and demand, ultimately resulting in heart failure. However, exercise can help optimize this condition by improving cardiac function. MI: Myocardial infarction; ACM: Arrhythmogenic cardiomyopathy; RIHD: Radiation-induced heart disease.

This review synthesizes recent research on myocardial energy metabolism in both physiological and pathological conditions, with a focus on the effects of exercise on myocardial metabolism and mitochondrial function. Ultimately, the goal is to provide insights that may guide the clinical application of exercise in cardiac rehabilitation. The key points of this review are summarized in Figure 1.

## Heart and myocardial metabolism

### Myocardial metabolism under physiological conditions

The heart continuously generates ATP to meet its high energy demands, primarily through mitochondrial oxidative phosphorylation. Under normal conditions, approximately 60%–70% of ATP comes from fatty acid (FA) oxidation, 10%–30% from glucose metabolism, with smaller contributions from ketone bodies, lactate, and amino acids [2, 4].

#### FA metabolism

FAs serve as the heart’s primary energy source under physiological conditions. The heart acquires FAs from two main sources: free FAs (FFAs) released through triglyceride (TG) hydrolysis by lipoprotein lipase and non-esterified FAs (NEFAs) bound to albumin [2, 5]. These FAs are transported into cardiomyocytes via proteins such as CD36, where they undergo β-oxidation to generate acetyl-CoA. Acetyl-CoA then enters the TCA cycle to produce ATP [2, 5, 6].

#### Glucose metabolism

Glucose is absorbed via GLUT1 and GLUT4 transporters, with GLUT4 being the predominant transporter in adults [7–9]. After uptake, glucose primarily undergoes glycolysis, yielding pyruvate or lactate. Additionally, auxiliary pathways, such as the hexosamine and pentose phosphate pathways contribute to redox balance and biosynthesis, providing key intermediates for cellular functions [2, 6].

#### Ketone body metabolism

Ketone bodies in the body are primarily synthesized from CoA, which is generated through the oxidation of FAs in the liver. Among these, β-hydroxybutyrate (βOHB) is the predominant ketone body oxidized in the heart.

βOHB enters cardiac cells via the monocarboxylate transporter 1 (MCT1) and is subsequently oxidized in the mitochondria [2, 8]. There, βOHB dehydrogenase 1 (BDH1) converts βOHB into acetoacetate, which is then activated into acetoacetyl-CoA by succinyl-CoA transferase (SCOT). Acetoacetyl-CoA undergoes thiolysis to produce acetyl-CoA, which enters the TCA cycle to generate ATP [2, 10].

#### Branched chain amino acid metabolism

Branched-chain amino acids (BCAAs)—including leucine, isoleucine, and valine—are essential amino acids primarily obtained through the diet and, to a lesser extent, synthesized *de novo* by gut microbiota [11]. BCAAs undergo reversible transamination, mediated by mitochondrial BCAA transaminase (BCAT2), to produce branched-chain α-keto acids (BCKAs) and glutamate. BCKAs then undergo irreversible decarboxylation, catalyzed by the BCKA dehydrogenase (BCKDH) complex, ultimately leading to their catabolism into acetyl-CoA and succinyl-CoA. These metabolites enter the TCA cycle or contribute to cellular biosynthetic pathways through anaplerosis [11]. The activity of BCKDH is regulated through inhibitory phosphorylation by BCKDH kinase (BCKDK) and activating dephosphorylation by mitochondrial protein phosphatase 2C family (PP2Cm), while BCKAs can also allosterically inhibit BCKDK [12].

Although BCAA oxidation contributes less than 2% of the heart’s total energy supply [8], BCAAs play significant metabolic roles in other physiological contexts. Notably, the mammalian target of rapamycin (mTOR) signaling pathway—a key regulator of protein synthesis and cellular growth—is influenced by BCAAs, particularly leucine. Activation of the mTOR pathway can also trigger metabolic processes that reduce insulin sensitivity [13]. As a result, BCAAs are closely linked to insulin resistance.

#### Substrate interactions under physiological conditions

Myocardial energy production relies on glucose and FA oxidation, which interact competitively through the Randle cycle [14, 15]. Under normal conditions, FA oxidation predominates, inhibiting glucose metabolism [16]. Conversely, increased glucose oxidation can suppress FA oxidation, a process regulated by hormonal signals like insulin [17]. This competition enables the heart to adjust its substrate use based on availability and physiological demands. Additionally, ketone bodies and BCAAs serve as alternative substrates, further influencing the balance between glucose and FA metabolism [17, 18].

### Myocardial metabolism during HF

HF is a clinical syndrome characterized by shortness of breath and limited physical activity due to impaired ventricular filling or ejection [2]. It is classified based on left ventricular ejection fraction (LVEF) as follows: HFrEF (LVEF < 40%), HFmrEF (LVEF 40%–49%), and HFpEF (LVEF ≥ 50%) [19].

This classification provides a framework for understanding variations in pathophysiology and treatment responses. In HF, the heart adapts its energy pathways based on workload, substrate availability, and hormonal status [20, 21]. Research suggests that during HF onset and progression, adaptive or maladaptive changes in myocardial metabolism typically precede alterations in cardiac function [10, 22, 23]. Notably, during the decompensated phase, ATP production declines by 30%, creating an energy supply–demand imbalance that ultimately contributes to HF progression [23, 24]. This underscores the critical role of energy metabolism in HF. In the following section, we summarize the specific metabolic changes occurring in the myocardium during HF.

#### FA metabolism

As HF progresses, the overall energy metabolism of the myocardium gradually declines. Impaired FA oxidation has been observed in both human and animal models of HFrEF [2, 25, 26]. In animal models, this decline in FA oxidation may be linked to the downregulation of peroxisome proliferator-activated receptor-α (PPAR-α) and peroxisome proliferator-activated receptor-γ coactivator 1 (PGC-1) signaling [6, 27]. However, whether these mechanisms also apply to humans remains unknown.

The phenomenon of reduced FA oxidation is not always consistent. During the compensation phase of HF, FA uptake and oxidation do not decrease [28], and in congestive HF, cardiac FA uptake actually increases [29]. These variations in FA utilization may depend on the type, severity, and progression of the disease. Additionally, myocardial FA oxidation is elevated in obesity and type 2 diabetes, likely due to glucose utilization disorders, insulin resistance, and other factors [30, 31]. The FA transporter protein CD36, a downstream target of PPAR-α, can be upregulated by PPAR-α overexpression [32]. Interestingly, CD36 knockout accelerates HF progression in cases of pressure overload but delays it in diabetic cardiomyopathy. These findings underscore the complexity of FA metabolism in different pathological states, highlighting the need for further in-depth research.

Increased FFA concentrations are strongly correlated with a higher risk of HF [33, 34]. Disruptions in myocardial FA metabolism can lead to lipid accumulation within cardiomyocytes, impairing their function and metabolism [35–37]. Specifically, palmitic acid accumulation alters cardiomyocyte membranes, increases oxidative stress, and disrupts homeostasis, ultimately causing mitochondrial dysfunction, apoptosis, and insulin resistance—hallmarks of cardiac lipotoxicity [38–41]. Additionally, acyl-CoA, a key molecule in lipid metabolism, is found to be reduced in failing human hearts and pressure-overloaded animal models. LVAD treatment can restore acyl-CoA levels through mechanical unloading, benefiting cardiac function. Similarly, in pressure-overload animal models, overexpression of acyl-CoA synthetase-1 (ACSL1) replenishes acyl-CoA levels, reduces lipotoxic ceramide species like palmitoyl-ceramide, and shifts the ceramide profile, thereby mitigating cardiac lipotoxicity [42]. Although some studies suggest that ACSL1 overexpression can promote lipid accumulation and exacerbate heart lipotoxicity, in pressure-overloaded hearts, ACSL1-mediated restoration of acyl-CoA levels significantly counteracts maladaptive lipid profile changes. This protective effect outweighs potential baseline risks under oxidative stress [43].

#### Glucose metabolism

To compensate for reduced FA oxidation, cardiomyocytes typically enhance glucose metabolism. However, even with increased glucose uptake and utilization, glucose metabolism may still be insufficient to fully offset the decline in FA oxidation, leading to an overall reduction in energy output. This energy deficit underpins the metabolic vulnerability of the failing heart. A decline in mitochondrial function is an early hallmark of cardiac hypertrophy, which accelerates the progression of HF [44]. In failing hearts, the uncoupling of glycolysis from glucose oxidation reduces energy efficiency, leading to increased proton production and the accumulation of glycolytic intermediates, particularly phosphoglycerate, which is strongly associated with HF risk [45–47]. Interestingly, a study using a canine pacing-induced HF model observed an increase in glucose oxidation [48], suggesting that myocardial glucose metabolism may vary depending on the experimental model or disease context. This discrepancy could be related to differences in disease severity or shifts in myocardial metabolic pathways.

GLUT1 upregulation in ischemic hearts disrupts energy homeostasis and exacerbates HF [49]. This upregulation has also been observed in pressure-overload models, where pressure overload activates the nuclear effector Yes-associated protein 1 (YAP). In cardiomyocytes, YAP interacts with TEAD1 and HIF-1α to upregulate GLUT1 and activates glycolysis, ultimately disrupting energy homeostasis. The accumulation of glycolytic intermediates promotes ventricular remodeling [50]. Conversely, pressure overload suppresses GLUT4 expression, impairing glucose uptake and potentially accelerating HF progression [51]. GLUT4 downregulation also increases endoplasmic reticulum stress and extracellular matrix (ECM) deposition, worsening ventricular remodeling after myocardial infarction. Moreover, glucose metabolism dysregulation can lead to the accumulation of advanced glycation end products (AGEs), which generate excessive reactive oxygen species (ROS), inducing oxidative stress [52]. This oxidative stress further damages cardiomyocytes and compromises cardiac function.

Interestingly, in diabetic cardiomyopathy, increased GLUT4 expression—intended to sustain myocardial glucose utilization—paradoxically accelerates mitochondrial dysfunction [53]. Thus, GLUT4’s role varies across pathological conditions and must be considered in context.

Under pathological conditions, glucose participates in non-energy-related signaling pathways such as the hexosamine biosynthetic pathway, pentose phosphate pathway, and carbon cycling, generating metabolites that disrupt metabolic balance. These pathways may contribute to cardiac hypertrophy and remodeling [54, 55]. Targeting these dysregulated pathways could help restore energy homeostasis and mitigate myocardial remodeling [56].

#### Ketone body metabolism

Ketone body metabolism in failing hearts is generally considered a compensatory mechanism for the reduced oxidation of other metabolic substrates. A substantial body of high-quality research has shown that circulating ketone body levels are elevated in HF patients and that ketone body utilization in the heart is also enhanced. This finding has been consistently demonstrated in animal models [57–61]. This phenomenon occurs because ketone body utilization in the heart is directly proportional to its delivery [62].

Notably, circulating ketone body levels are not elevated in HFpEF patients [63]. In recent years, numerous studies have indicated that higher circulating ketone body levels are strongly correlated with worsening HF or adverse outcomes in HFrEF. Consequently, its potential as a clinical predictive biomarker is gaining increasing attention [64–68].

In BDH1^−/−^ or SCOT^−/−^ animal models, impaired myocardial ketone utilization exacerbates oxidative stress, induces mitochondrial dysfunction, and disrupts myofibril ultrastructure, ultimately rendering the heart incapable of handling stress overload or ischemia. Consequently, this accelerates the progression of cardiac decline. However, exogenous βOHB supplementation has been shown to mitigate pathological ventricular remodeling and slow disease progression [69, 70].

βOHB supplementation within physiological concentration ranges in chronic HFrEF patients has been shown to enhance cardiac output [71]. Increasing circulating ketone levels as a potential HF therapy is an active area of translational research, with approaches including ketone infusion, ketone ester (KE) administration, and ketogenic diets, as detailed in Table 1. While exogenous ketone therapy benefits the heart, prolonged exposure to a ketone-rich environment may have adverse effects, such as v-ATPase proton pump degradation, contractile dysfunction, and insulin resistance [72]. Similarly, excessive βOHB accumulation may disrupt metabolic balance, exacerbating cardiac dysfunction over time. An *ex vivo* study on low-flow perfused hearts also found that increased βOHB accumulation impaired cardiac contractile recovery [73]. These findings underscore the complexity of ketone body roles in different pathological states, with unclear long-term effects and a potential risk of impaired cardiac function.

**Table 1:** Evidence supporting the cardiovascular advantages of supplementing ketones

**Form**	**Disease**	**Object**	**Key discoveries**	**Reference**
KE (0.5 g/kg)	CS	Human	CO*↑*; Tissue oxygenation*↑*; Glycemic*↓*	[204]
KE (395 mg/kg)	COVID-19	Human	GLS*↑*	[205]
KE (0.38 g/mL)	TAC	Mice	Cardiomyocyte hypertrophy*↓*; Elevated cardiac periostin*↓*; CO*↑*; Fibrosis*↓*	[206]
KE (20%)	DCM	Mice	SCOT*↑*; BDH1*↑*; ACAT1*↑*; ROS*↓*; Complex-II*↑*; Complex-IV*↑*Complex-V*↑*	[207]
KE (550 mg/Kg, 2 occ/d)	AMI	Pig	Inflammation*↓*; Apoptosis*↓*; Oxidative stress*↓*	[208]
KE (10%/15%)	TAC/MI post-MI HF	Mice Rat	LVEF*↓*; Fibrosis*↓*; Cardiomyocyte hypertrophy*↓*; SCOT*↑*	[209]
KE (25 g/occ, 4 occ/d, 14 d)	HFrEF	Human	CO*↑*; LVEF*↑*; FP*↓*; NT-proBNP*↓*; Cardiac volumes*↓*	[210]
TRF	BDH1^−/−^	Mice	Ventricular remodeling*↓*; Mitochondrial bioenergetics*↑*	[211]
Ketogenic diet (10% protein, 90% fat, 5d)	Trx1 KD	PCMs Mice	Trx1*↑*; Oxidative stress*↓*; Ventricular remodeling*↓*	[212]
Ketogenic diet (10.4 kcal protein, 0.1 kcal carbohydrates, 89.5 kcal fat)	TAC	Mice MCEC	Angiogenesis*↑*	[213]
SCOT^−/−^ in skeletal muscle	TAC	Mice	NLPR3*↓*; Inflammation*↓*	[214]
D-βOHB/L-βOHB (0.36 mg/kg)	Normal	Pig	Coronary artery dilation*↑*; Afterload*↓*; CO*↑*	[215]
βOHB (10 mmol/kg, 1 occ/w, 15 w)	HFpEF	Mice	NOX2/GSK-3β ↓; Treg cell*↑*; Inflammation*↓*; Ventricular remodeling*↓*	[216]
βOHB (483 mg/kg, 4 occ/h)	Normal	Human	CO*↑*	[217]
βOHB (160, 200, 240 mg/kg/d; 10 w)	Diabetes	Rat HCMECs	COL4*↓*; Cu/Zn-SOD*↑*; NT*↓*; Microvascular fibrosis*↓*	[218]
βOHB (10 mmol/kg)	I/R	Mice	mTOR pathway(−); Mitophagy*↑*; Infarct size*↓*; Oxidative stress*↓*	[219]
βOHB (10 mmol/kg/d, 5 occ/d, 5 d)	DOX cardiotoxicity	C57BL/6 H9C2	Fibrosis*↓*; apoptosis*↓*; Oxidative stress*↓*; Mitochondrial membrane integrity*↑*	[220]
βOHB (360 mg/kg/h)	Heart transplantation	Pig	SV*↑*; Arterial elastance*↓*; CO*↑*; dP/dt*↑*	[221]
D-βOHB (3 mg/g, 1 occ/d)	Septic cardiomyopathy	Mice H9C2 cell	FoxO3a/MT2*↑*; Mitochondrial dysfunction*↓*; ROS*↓*	[222]
Cardiac perfusion βOHB (3/10 mM)	Normal	Rat	CO*↑*; LVEF*↑*; SV*↑*; dP/dt_max_ *↑*; Vascular resistance*↓*	[223]

CS: Cardiogenic shock; CO: Cardiac output; KE: Ketone ester; TRF: Time-restricted feeding; AMI: Acute myocardial infarction; I/R: Ischemia/reperfusion; CHFrEF: Chronic heart failure with reduced ejection fraction; HFrEF: Heart failure with reduced ejection fraction; HFpEF: Heart failure with preserved ejection fraction; SV: Stroke volume; HR: Heart rate; LVEF: Left ventricular ejection fraction; FP: Filling pressure; GLS: Global longitudinal strain; TRF: Time-restricted feeding; PCMs: Primary cardiomyocytes; TAC: Transverse aortic constriction; DCM: Diabetic cardiomyopathy; CS: Citrate synthase; EMPA: Empagliflozin; DAPA: Dapagliflozin; SOTA: Sotagliflozin; LVDF: Left ventricular diastolic function; LVV: Left ventricular volume; LVM: Left ventricular mass; LVSF: Left ventricular systolic function; DBP: Diastolic blood pressure; SBP: Systolic blood pressure.

#### BCAA metabolism

Recent studies have identified elevated circulating BCAA levels as an independent predictor of cardiovascular events, including HF onset, plaque rupture, and thrombus formation—factors that can contribute to ischemic cardiomyopathy and poor clinical outcomes [74–79]. This correlation highlights the critical role of BCAA dysregulation in cardiovascular pathology. In animal models of pressure overload and myocardial infarction, BCAA metabolism was found to be downregulated in both the compensated and decompensated phases of HF. Interestingly, while BCAA levels increased in heart tissue, they did not rise in plasma, suggesting localized metabolic alterations [80, 81].

To better understand how BCAAs influence HF, various experimental models have been used to examine their effects on cardiac metabolism and function. In one study, pressure-overloaded mice fed a BCAA-rich diet exhibited increased histone H3K23 propionylation (H3K23Pr) at promoters, downregulation of electron transport chain complexes (ETC I–V), reduced mitochondrial respiration, increased myocardial fibrosis, and worsened cardiac function. Conversely, a BCAA-deficient diet produced the opposite, beneficial effects [82]. These findings suggest that excess BCAAs disrupt mitochondrial energy metabolism and contribute to cardiac dysfunction under pathological conditions.

Meanwhile, a multi-omics study found that the downregulation of BCAA metabolism does not occur in endurance exercise-induced physiological cardiac hypertrophy, indicating that it is a distinct feature of pathological cardiac hypertrophy [83]. Interestingly, another study showed that while BCAA metabolic defects do not drive HF progression, BT2—a potent inhibitor of BCKDK—enhances systemic BCAA metabolism, leading to reduced vascular tension, lower blood pressure, and ultimately cardioprotective effects by preventing adverse cardiac remodeling [84].

On the other hand, as mentioned earlier, activation of the mTOR pathway can reduce insulin sensitivity and contribute to insulin resistance. BCAAs can also impair insulin-stimulated glucose uptake in skeletal muscle and inhibit insulin-induced phosphatidylinositol 3-kinase activity, leading to impaired glucose uptake, insulin resistance, and subsequent metabolic disorders [85]. However, paradoxical effects of BCAA metabolism have been observed in different contexts. In BCATm^−/−^ animal models, elevated circulating BCAA levels were associated with beneficial phenotypes, such as reduced obesity and increased insulin sensitivity [86]. Furthermore, studies suggest that BCKA, rather than BCAA, is the key mediator of cardiac insulin resistance and could serve as a target for modifying cardiac insulin sensitivity [87]. In a study on pressure-overloaded PP2Cm^−/−^ mouse models, Krüppel-like factor 15 (KLF15) was identified as a key upstream regulator of reduced cardiac BCAA catabolism. Its loss or inhibition led to defective BCAA catabolism, resulting in elevated BCKA levels, increased superoxide production, oxidative stress damage, and worsened cardiac function [88]. Another study on BCKDH knockout/overexpression mice found that BCKDH knockout caused increased BCKA levels, suppressed insulin-induced AKT phosphorylation, and reduced glucose uptake. In contrast, BCKDH overexpression led to beneficial cardiac outcomes, reversing the detrimental effects observed in knockout models [89, 90]. Collectively, these animal studies demonstrate that BCKA, rather than BCAA, plays a central role in mediating cardiac insulin resistance. Human studies have also identified elevated serum BCAA levels as a metabolic hallmark of insulin resistance, with gut microbial species, such as Prevotella copri and Bacteroides vulgatus emerging as key drivers of the link between BCAA biosynthesis and insulin resistance [91].

## Effects of exercise on myocardial energy metabolism

Physical activity encompasses any bodily movement that increases energy expenditure due to skeletal muscle contraction, typically exceeding a resting metabolic rate of 3.5 mL O_2_/min/kg or one metabolic equivalent (MET) [92]. Exercise, a specific subset of physical activity, consists of planned, structured, and repetitive movements designed to maintain or enhance physical health [93]. Based on skeletal muscle involvement, exercise can be classified as either dynamic or static. Dynamic exercise includes endurance activities that involve continuous muscle contractions, such as swimming, jogging, and walking. A key characteristic of dynamic exercise is the proportion of maximal oxygen uptake it requires, which can contribute to eccentric ventricular remodeling. In contrast, static exercise involves sustained muscle contractions to overcome resistance, as seen in weightlifting and deadlifts. Its primary defining factor is the percentage of maximum voluntary contraction, which may lead to concentric ventricular remodeling [92, 94].

### Exercise and myocardial metabolism

During physical activity, a physiological stimulant, the heart’s contractility and oxygen consumption can rise up to ten times their resting levels [95]. The increased cardiac workload during exercise promotes metabolic flexibility in substrate utilization, particularly affecting FA and lactate metabolism [95–97]. Specifically, exercise stimulates catecholamine-driven fat metabolism, elevating circulating FFAs to approximately 2.4 mM—6–10 times their resting levels [95, 98]. At this stage, FA metabolism is primarily driven by oxidative processes for energy supply rather than biosynthesis, reducing lipid accumulation and, consequently, the risk of lipotoxicity [99]. Additionally, the increase in FA oxidation, along with enhanced mitochondrial cristae density during exercise, contributes to beneficial physiological cardiac hypertrophy, further strengthening the protective effects of exercise on cardiac function [100].

During exercise, the extensive contraction of skeletal muscles significantly increases circulating lactate concentrations, approaching 10 mM [101, 102]. Meanwhile, the heart is a major consumer of lactate, with its utilization rate positively correlated with circulating lactate levels [3]. Interestingly, elevated lactate levels are also associated with increased FA oxidation, suggesting a synergistic effect that enhances the heart’s overall energy supply under high-load conditions. Additionally, the reduction in glucose utilization appears to coincide with increased FA and lactate use, likely due to substrate competition and the heart’s adaptation to sustained physical demand [103]. Studies further indicate that while acute exercise boosts lactate utilization in the heart, long-term exercise has little effect on lactate oxidation capacity or lactate dehydrogenase content. However, it does enhance FA oxidation and transport [104, 105].

Additionally, long-term exercise can enhance cardiovascular health by reducing the activity of 6-phosphofructo-2-kinase/fructose-2,6-bisphosphatase 2 (PFKB2), an enzyme that suppresses glycolysis and decreases glucose utilization, thereby promoting physiological ventricular remodeling [106]. Furthermore, a study on female rats fed a high-fat, high-sugar (HFHS) diet found that, even eight weeks after exercise cessation, prior exercise corrected the redox imbalance caused by the HFHS diet and restored mitochondrial efficiency [107]. This suggests that the benefits of exercise persist even after training ends. Understanding these general metabolic adaptations provides a foundation for exploring how different exercise types and intensities uniquely affect myocardial energy metabolism.

### The effect of exercise type and intensity on myocardial metabolism

Different exercise intensities lead to varying cardiac metabolic responses. During low- to moderate-intensity exercise, both male and female mice exhibit increased circulating levels of ketones and lactate. However, during high-intensity exercise, male mice show elevated lactate levels without a corresponding rise in ketones, whereas female mice experience an increase in both metabolites [108]. This suggests that female mice have a more adaptable myocardial metabolism in response to physiological stressors like exercise.

A 12-week exercise program study involving 52 healthy participants found that high-intensity interval training (HIIT) and combined training (CT) increased cardiolipin levels and tricarboxylic acid cycle metabolites, indicating enhanced mitochondrial activity from aerobic training. In contrast, resistance training (RT) increased plasma membrane phospholipids, highlighting its role in preserving cellular integrity. Notably, all three exercise types improved insulin sensitivity and cardiac metabolic markers [109].

These findings underscore the heart’s remarkable ability to adapt its energy substrate selection based on various factors, including the type, frequency, intensity, duration, and form of exercise, as well as gender differences. Additionally, genotypes that regulate myocardial metabolism may serve as markers to differentiate physiological ventricular remodeling due to exercise from pathological remodeling caused by disease stimuli [110]. This emphasizes the potential clinical value of exercise as a non-pharmacological strategy for protecting cardiovascular health.

### The harmful effects of exhaustive exercise (EE) on myocardial metabolism

EE refers to prolonged, high-intensity physical activity that surpasses the body’s tolerance [111]. It has been extensively studied in both human and animal models, with evidence showing that EE can lead to adverse cardiovascular remodeling, including myocardial ultrastructural damage, myocardial fibrosis, malignant arrhythmias, arterial stiffening (aortic and carotid), and elastic lamina rupture [112–115].

Increasing evidence suggests that inflammation and oxidative stress are key mechanisms through which EE induces harmful cardiovascular remodeling [116–119]. For example, EE can elevate inflammatory factors, such as IL-6, IL-10, and TNF-α, activate inflammatory signaling pathways like NF-κB [120], and significantly increase ROS levels by disrupting cellular redox balance [121]. Elevated ROS damages mitochondria by breaking mtRNA and causing genome mismatches. It also disrupts mitochondrial membranes, leading to Ca^2^^+^ overload and further redox imbalance [121]. These changes collectively contribute to the progressive deterioration of mitochondrial structure and function.

EE also downregulates the calcium-binding protein S100A1, weakening its regulatory effect on PGC-1α. This suppression reduces the expression of key proteins involved in mitochondrial biogenesis and energy metabolism, such as Ant1 and Tfam, ultimately leading to mitochondrial dysfunction and metabolic imbalance [122]. Additionally, by inhibiting the PGC-1α/Complex I/II pathway, EE decreases mtRNA expression, upregulates mitochondrial fission-related proteins (e.g., Drp1 and Fis1), and suppresses mitochondrial fusion-related proteins (e.g., Mfn2 and OPA1), disrupting mitochondrial fission–fusion dynamics [123]. This imbalance further exacerbates oxidative stress, activates inflammatory pathways, and inhibits the Nrf2/HO-1 antioxidant mechanism, creating a vicious cycle that ultimately results in cardiac metabolic dysregulation [118, 124, 125].

As previously mentioned, GLUT4 is the most abundant glucose transporter in the myocardium. Under physiological conditions, it is primarily localized within intracellular vesicular structures (e.g., the Golgi apparatus and microsomes). However, in response to ischemia-hypoxia, insulin, or muscle contraction, GLUT4 translocates to the plasma membrane to facilitate glucose transport. Studies have shown that exhaustive exercise (EE) negatively correlates with GLUT4 translocation, disrupting the balance between glucose supply and demand in myocardial cells and exacerbating myocardial injury [126]. AMPK plays a key role in exercise-induced mechanotransduction, promoting myocardial cell autophagy, enhancing mitochondrial biogenesis, and exerting cardioprotective effects [127]. However, the same study found that EE has limited effects on AMPK activation, and its associated cardioprotective mechanisms are insufficient to counteract the cardiac damage caused by EE. In contrast, moderate aerobic exercise can mitigate these adverse effects. Notably, in an obese animal model, EE was found to inhibit AMPK activation [128], whereas moderate aerobic exercise provided beneficial cardioprotective effects [129]. This suggests that EE may contribute to AMPK dysregulation, reducing its ability to maintain energy homeostasis, impairing GLUT4 translocation, and exacerbating metabolic stress.

Additionally, metabolomics studies have shown that EE induces significant changes in lipid and amino acid metabolism, including a marked increase in metabolites, such as glutamate, glutamine, arachidonic acid, and myostatin. The accumulation of these substances may upregulate the PTGS2/MAOB pathway, further promoting myocardial cell inflammation and exacerbating myocardial damage [130].

Despite these findings, the effects of EE across different age groups and exercise intensities remain unclear. Future studies should establish exercise guidelines for various populations to optimize cardiac rehabilitation and safeguard cardiovascular health.

## The connection between myocardial energy metabolism, exercise, and HF

In HF, cardiac metabolic adaptability declines, and energy production becomes restricted, further exacerbating metabolic disturbances and mitochondrial dysfunction in cardiomyocytes [5]. These impairments are a hallmark of HF progression, highlighting the need for targeted therapeutic strategies. Exercise, a safe and cost-effective intervention, has been shown to enhance myocardial metabolism, regulate metabolic protein expression, and reduce cardiovascular risks across various physiological conditions [131, 132]. Additionally, exercise improves energy efficiency and restores mitochondrial health, making it a promising therapeutic approach. Numerous animal and human studies have explored the effects of different types of exercise on heart health. Table 2 summarizes the latest evidence on the cardiovascular benefits of exercise.

**Table 2:** Evidence for the cardiovascular benefits of exercise

**Form of exercise**	**Rate and strength**	**Disease**	**Key discoveries**	**Reference**
Voluntary wheel running (8 w)	–	HFD	Bodyweight*↓*; Insulin resistance*↓*; Mitochondrial dysfunction*↓*; White fat browning*↑*	[224]
Treadmill exercise (5 d/w, 4 w)	10–15 m/min, 60 min/d	T1D	Glucose transport*↓*; Ketone body metabolism*↓*; FA metabolism*↑*; Insulin resistance*↑*	[225]
Swimming exercise (5 d/w, 12w)	80% of the critical load intensity, 30min/d	OVX	CD36*↑*; GLUT4*↑*; TG*↓*	[226]
Treadmill exercise (5 d/w, 4 w)	60%–75% Vmax, 60 min/d	OVX+MI	Decreased pro-inflammatory cytokines*↓*; Inflammation*↓*; IL-10*↑*; Dimethylamine*↓*	[227]
Treadmill exercise (5 d/w, 12w)	50%–60% V_max_, 60min/d	OVX	Nox4*↓*; SERCA2*↑*; Mitochondrial dysfunction*↓*; Myocardial contractility*↑*	[228]
Treadmill exercise (5 d/w, 9 w)	30 min/d, 15 m/min	LPS model	NO*↓*; TNF-α ↓; IL-1β ↓; CRP*↓*; CAT*↑*; Apoptosis*↓*	[229]
Treadmill exercise (4 d/w, 8 w)	50%–75% Vmax, 60 min/d	HFrD model	p-p70S6K*↑*; p-ERK*↑*; IRβ-PI3K-AKT pathway activation	[230]
Treadmill exercise (5 d/w, 8w)	HIIT: (6–12)×2min, 2–6 m/min	T2DCM	B-catenin*↓*; c-Myc*↓*; GSK3B*↑*; Apoptosis*↓*; Fibrosis*↓*	[231]
Treadmill exercise (3-5 d/w, 8 w)	MICT: 60% V_O2max_, 60 min/d; 40%–50% V_O2max_ 10min HIIT: 90% V_O2max_, 4×4 min; 60% V_O2max_ 3min	Advanced HFpEF	Ca^2+^ leak*↓*; SV*↑*	[232]
Treadmill exercise (5 d/w, 20 w)	30–50 min/d, 10–15 m/min	HFpEF	H_2_S*↑*; Apoptosis*↓*; Insulin resistance*↓*; Diastolic function*↑*; BGC*↓*	[233]
Treadmill exercise (3 d/w, 8 w)	65% V_max_, 60 min/d	AICM	Nrf2/Keap1/HO1 pathway activation; Apoptosis*↓*	[234]
Cycling exercise (12 w, 3 d/w)	MICT and HIIT: 4w−400kJ+8w−300kJ MICT: 60% V_O2max_, 61 min HIIT: 90% V_O2max_, 9×4 min/occ SIT: 100% V_O2max_, 80×6 s/occ	Overweight women	V_O2max_ *↑*; Body weight*↓*; Insulin sensitivity*↑*	[235]
Exercise (12 w, 3 d/w)	50%–70%V_O2max_, 35–40 min	Postmenopausal women	LDL*↓*; TG*↓*; HgbA1c*↓*; IGF-1*↑*	[236]
Exercise (16 w, 3 d/w)	43 min	MetS	Body weight*↓*; Waist circumference*↓*; MAP*↓*	[237]
Running exercise (8 w, 3 d/w)	MICT: 60%–75% V_O2max_, 3500–5000 m HIIT: 85%–100% V_O2max_, 7×200–10×200 m	Obesity	BMI*↓*; Visceral fat*↓*; SBP*↓*; TC*↓*; BGC*↓*; TG*↓*s	[238]
Running exercise (3 d/w, 12 w)	AIT: Warm-up 50%–60% V_O2max_ 10min; Exercise: 90%–95% V_O2max_ 4 min, 4 occ; 50%–70% V_O2max_ 3 min, 4 occ; 38 min/occ MIT: 70%–75% V_O2max_, 47 min/occ	post-MI heart failure	BNP*↓*; LVEDV*↓*; LVESV*↓*; BNP*↓*; Vasodilation	[239]
Cycling exercise (3 d/w, 12 w)	MICT: 60%–75% HRmax, 45 min/occ; HIIT: 90%–100% HRmax, ex 1 min, 12 occ, 2 occ/w; 90%–95% HRmax, 4 min, 8 occ, 1 occ/w	PCOS	V_O2max_ *↑*; Insulin sensitivity*↑*; Aerobic capacity*↑*	[240]
Running exercise (3 d/w, 10 w)	70% HRmax, 40 min/occ;	PMW	Body weight*↓*; Fat mass*↓*; Resting glucose*↓*; HbA1c*↓*; V_O2max_ *↑*	[241]
Running exercise (3 d/w, 12 w)	MICT: 30% HRmax, 5 min/occ; 65%–75% HRmax, 35 min/occ HIIT: 50%–60% HRmax, 10 min; 85%–95% HRmax, exercise 1 min, 10 occ	T2D	HbA1c*↓*; BDNF*↑*; Blood lipids*↓*; Blood glucose*↓*	[242]
Running exercise /Cycling exercise (3 d/w, 12 w)	RPE12-14, ≥ 20 min, 1 w; HIIT: RPE15-17, 2–4 min/occ, 5–8 occ; MICT: RPE12-14, 20–45 min/occ	MI+MetS	Waist circumference*↓*; Blood glucose*↓*; TG*↓*; DBP*↓*	[243]
Running exercise (12–16 w, 2 d/w)	MICT: 35%–50% HRmax, 10 min/occ; Low-HIIT: 35%–50% HRmax, 19 min/occ; 80%–90% HRmax, 4 min/occ; High-HIIT; 35%–50% HRmax, 40 min/occ; 80%–90% HRmax, 4 min/occ, 4 occ;	MetS	Leukocyte counts*↓*; TG*↓*; V_O2max_ *↑*	[244]
Cycling exercise (3 w, 5 d/w)	30 min, 2 occ/d	HFrEF	MECKI*↑*; CVE incidence*↓*	[245]

MI: Myocardial infarction; OVX: Ovariectomy; LPS: Lipopolysaccharide; HF: Heart failure; PDC: Pyruvate dehydrogenase complex; GLUT: Glucose transporter; PGC-1: Peroxisome proliferator-activated receptor γ coactivator-1; CRP: C-reactive protein; BNP: Brain natriuretic; PAH: Pulmonary arterial hypertension; HFD: High-fat diet; T1D: Type 1 diabetes; HFrD: High-fructose diet; T2DCM: Type 2 diabetic cardiomyopathy; SV: Stroke volume; BGC: Blood glucose concentration; AICM: Alcohol-induced cardiomyopathy; MetS: Metabolic syndrome; MAP: Mean arterial pressure; TC: Total cholesterol; SBP: Systolic blood pressure; CVE incidence: Cardiovascular events incidence; PCOS: Polycystic ovary syndrome; PMW: Postmenopausal women; HRmax: Peak heart rate; DBP: Diastolic blood pressure; TG: Triglycerides.

In a rat model of high-fat coronary artery disease, four weeks of treadmill exercise alleviated myocardial fibrosis, inflammation, and apoptosis by inhibiting NF-κB signaling. These effects collectively mitigated pathological remodeling and improved overall heart function [133]. In contrast, in a myocardial infarction model, 12 weeks of RT did not improve survival rates, myocardial structure, or function [134]. However, six weeks of moderate- or high-intensity endurance training in the same model significantly enhanced ATP production capacity and myocardial contractility [135]. Overall, these findings suggest that while exercise benefits the failing heart, its effectiveness depends on the type, duration, and intensity of training. As previously discussed, an imbalance in myocardial energy metabolism is a central contributor to HF. This section provides an overview of recent studies exploring how exercise modulates myocardial metabolism to support heart health.

### The influence of exercise on myocardial metabolism

Investigating how exercise-induced metabolic alterations in cardiomyocytes contribute to cardiovascular benefits remains a key area of research. In a study involving 50 patients with HFpEF (NYHA class II and III), a four-week cardiac rehabilitation program increased Sirt1 activity and βOHB levels while reducing oxidative stress. These changes correlated with higher NAD levels, an improved NAD/NADH ratio, and lower Ox-LDL [136]. Similarly, in a mouse model of coronary heart disease induced by a high-fat diet, an eight-week swimming regimen (55-min sessions, five days a week) downregulated miR-344g-5p, which targets HMGCS2. This inhibition of ketogenesis reduced lipid accumulation, thereby attenuating lipotoxicity-induced myocardial fibrosis, cardiomyocyte apoptosis, and subsequent cardiac dysfunction [137]. Building on these findings, another study explored whether different forms of exercise activate distinct molecular pathways to achieve similar cardiovascular protection. Voluntary wheel running (60 min per day for eight weeks) was shown to activate the AMPK/PGC1α pathway, enhance mitochondrial phosphorylation, reduce oxidative stress, shift metabolism from FA to glucose oxidation, correct metabolic disturbances in diabetic cardiomyopathy, and ultimately improve cardiac function while mitigating the disease’s adverse effects [138]. Microsomal TG transfer protein (MTP) plays a crucial role in lipid metabolism by facilitating lipid transport, apolipoprotein B assembly, and the release of chylomicrons and VLDL. Endurance training has been shown to increase MTP expression in fruit flies, improving systemic lipid imbalances and high-fat diet-induced cardiac dysfunction [139]. Additionally, endurance exercise alleviates age-related diastolic dysfunction and mitochondrial impairments, enhances lipid metabolism, and improves survival rates [140, 141]. Collectively, these findings underscore the role of exercise in restoring metabolic balance in failing cardiac muscle, ultimately preserving heart function.

GLUT4 is a key protein in glucose uptake and transport. Previous studies have shown that endurance exercise increases GLUT4 content in diabetic myocardium, thereby improving glucose utilization disorders in diabetic cardiomyopathy [142]. Research indicates that short-term treadmill exercise (60-min sessions, once daily, five days a week, for two weeks) can reverse GLUT4 decline in a rat model of pulmonary arterial hypertension (PAH), regulating glucose metabolism and improving PAH-induced diastolic dysfunction [143].

**Figure 2.**
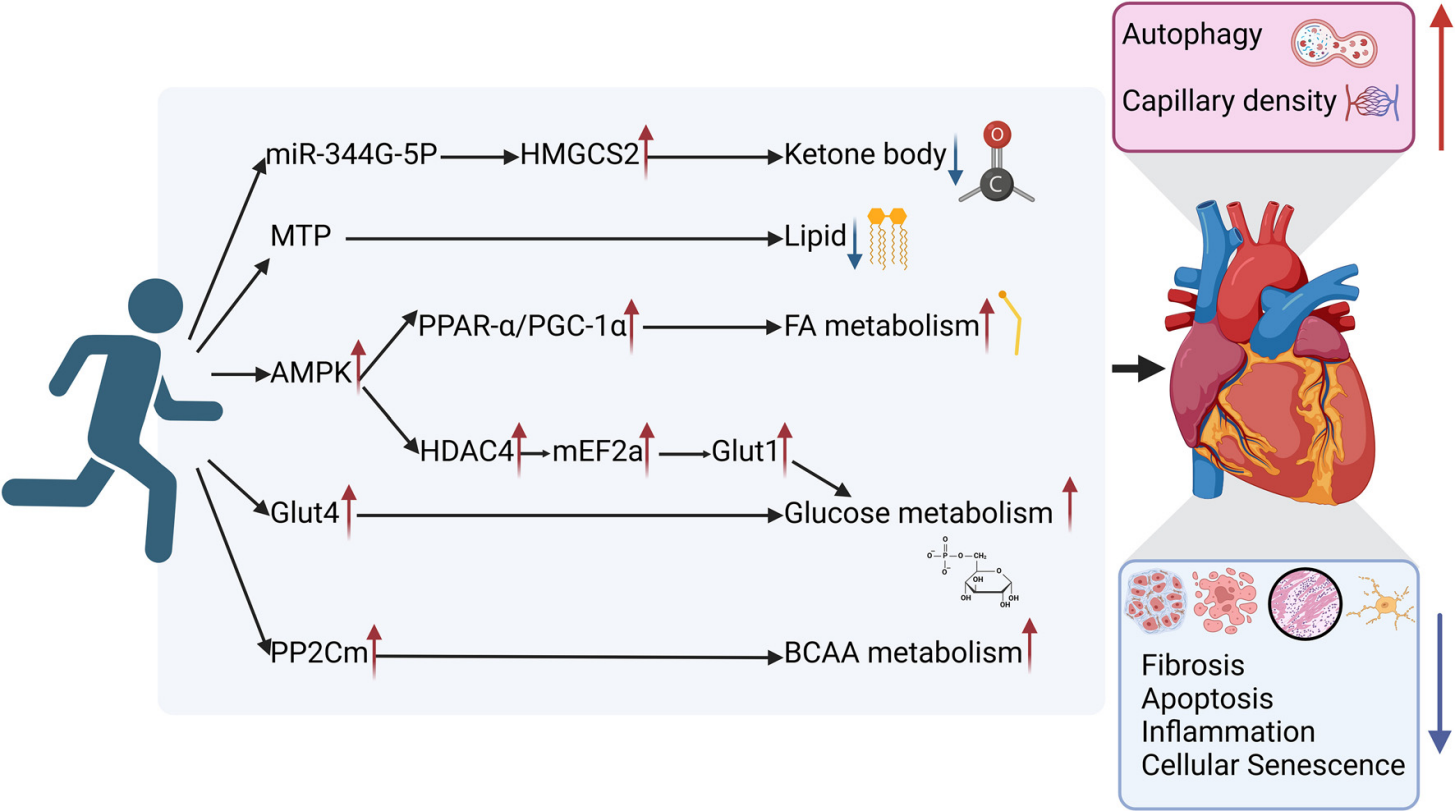
**Mechanisms of how exercise regulates myocardial metabolism and enhances heart function.** Exercise can activate various pathways in the body, thereby improving fatty acid metabolism, glucose metabolism, and BCAA metabolism, while also reducing ketone bodies and lipids. As a result, this ultimately leads to increased autophagy of cardiomyocytes and higher capillary density in the myocardium, which is accompanied by decreased aging, apoptosis, fibrosis, and inflammation in cardiomyocytes, collectively enhancing cardiac function. HMGCS2: 3-hydroxy-3-methylglutaryl-CoA synthase 2; MTP: Microsomal triglyceride transfer protein; PPAR-α: Peroxisome proliferator-activated receptor-alpha; HDAC4: Histone deacetylase 4; MEF2A: Myocyte enhancer factor 2A; GLUT1: Glucose transporter type 1; GLUT4: Glucose transporter type 4; PP2Cm: Protein phosphatase 2Cm; AMPK: AMP-activated protein kinase; BCAA: Branched-chain amino acid.

However, this short-term regimen did not reverse declines in FA and amino acid metabolism but did elevate PGC-1α and PPAR-γ, both linked to FA metabolism in HF [6, 30]. Longer treadmill training (60-min sessions, once daily, five days a week, for 12 weeks) restored GLUT4 levels in female mice, reducing cellular aging, inflammation, and oxidative stress while restoring autophagy and protecting cardiac function in high-fat diet-induced diabetes [144]. Beyond glucose metabolism, exercise significantly affects mitochondrial function through multiple pathways, with variability in outcomes likely influenced by animal models, disease conditions, and exercise protocols. Endurance training (60-min sessions, once daily, five days a week, for four weeks) stimulates myocardial AMPK, which phosphorylates histone deacetylase 4, reducing MEF2a inhibition in HF mice. This enhances GLUT1 expression, improves glucose metabolism, and strengthens cardiac function following myocardial infarction [145]. In a rat model of post-infarction HF, the same protocol provided cardiovascular benefits, increasing FA metabolism and reducing glycolysis, potentially through AMPK/PPAR-α pathway activation and mitochondrial function improvement [146, 147]. A study comparing exercise durations (15- vs 60-min sessions, once daily, five days a week, for eight weeks) found that low-intensity endurance exercise enhanced Sirt3 activity more effectively than higher-intensity training. This improved mitochondrial structure and autophagy in elderly post-infarction HF mice, reducing apoptosis, oxidative stress, and myocardial fibrosis while improving survival and cardiac function [148]. Overall, these findings emphasize the importance of specific exercise intensities and durations in mitigating mitochondrial dysfunction and promoting heart health in HF. Exercise enhances not only FA, glucose, and ketone metabolism in failing hearts but also boosts BCAA metabolism. This occurs via mitochondrial serine–threonine PP2Cm upregulation, reducing cardiac BCAA accumulation, myocardial fibrosis, apoptosis, and cardiomyocyte hypertrophy while increasing capillary density and LVEF, ultimately protecting the heart from ischemic damage [149]. Collectively, these studies underscore the role of tailored exercise in optimizing myocardial energy metabolism and alleviating HF (Figure 2).

### The influence of exercise on mitochondria

Building on the benefits of exercise-induced metabolic changes, the impact of physical activity on mitochondrial dynamics is a crucial factor in cardiovascular protection. As the primary site of metabolism, mitochondria are highly dynamic organelles that play a key role in cell survival and death during the progression of cardiovascular diseases [150]. Their dynamics—including biogenesis, fusion, fission, and autophagy—are essential for maintaining mitochondrial integrity, positioning, size, and function, all of which are critical for cardiovascular health [151]. Exercise actively influences mitochondrial shape and biogenesis, supports mitochondrial homeostasis, optimizes myocardial metabolic substrates, and enhances cardiovascular function [151–153]. While the precise mechanisms remain unclear [154], research suggests that treadmill running (60-min sessions, once daily, five days a week, for four weeks) activates the SIRT1/PGC-1α/PI3K/Akt pathway, enhances antioxidant defenses, and improves mitochondrial function, ultimately reducing myocardial fibrosis and enhancing heart function in aged rats following myocardial infarction [155]. The same treadmill regimen also increases AMPKα2 activity, boosts respiratory chain complex I function, promotes mitochondrial autophagy, and mitigates DOX-induced cardiac injury [156]. In the myocardium, mitochondria exist primarily as intermyofibrillar and subsarcolemmal (SS) populations. Exercise is particularly effective in regulating the redox balance and iron homeostasis of SS mitochondria, thereby contributing to overall mitochondrial stability [157].

HIIT, known for its time efficiency, is increasingly studied for its cardiovascular benefits compared to traditional exercise regimens like moderate-intensity continuous training (MICT). Mitochondrial function plays a key role in these protective mechanisms and has become a major focus of research. A randomized controlled trial involving obese individuals found that both MICT and HIIT improve mitochondrial respiratory function. However, HIIT was more effective in increasing mitochondrial numbers and enhancing cardiac contractility, underscoring its superior benefits for mitochondrial health [158, 159]. This suggests that different aerobic exercise intensities offer distinct mitochondrial advantages. Additionally, HIIT enhances mitochondrial size and morphology while reducing fragmentation caused by prolonged sitting, helping to preserve mitochondrial integrity [160].

Exercise influences not only mitochondrial dynamics—such as biogenesis, fusion, and fission—but also other critical aspects of mitochondrial health. Treadmill training (60-min sessions, once daily, five days a week, for 12 weeks) significantly increases mitochondrial-derived peptide levels in the heart, improving myocardial contraction and potentially enhancing diastolic function [161]. Similarly, treadmill running (45-min sessions, once daily, five days a week, for five weeks) boosts endothelial nitric oxide synthase (eNOS) activity in mitochondria, elevates nitric oxide production, enhances S-nitrosylation, reduces oxidative stress, improves mitochondrial function, and strengthens the heart’s ability to withstand ischemic hypoxia [162]. Beyond telomere maintenance, telomerase reverse transcriptase (TERT) in mitochondria plays additional roles. Voluntary wheel running (4350 ± 685 m per day, for three weeks) increases TERT activity in hearts under pressure overload, leading to improved complex I activity, enhanced antioxidant responses, and prevention of mitochondrial dysfunction—ultimately exerting an anti-hypertrophic effect [163].

Exercise has effects not only during performance but also afterward. Studies show that rats on a high-fat diet retain benefits such as enhanced mitochondrial respiratory efficiency, increased antioxidant response, and reduced inflammation for up to eight weeks after stopping exercise [107]. Genetic factors significantly influence the cardiac response to exercise. Under certain genetic conditions, exercise may fail to provide cardioprotective benefits. For instance, in a model of arrhythmogenic cardiomyopathy with a desmoglein-2 mutation, prolonged swimming sessions (90 min, five days a week, for 11 weeks) unexpectedly led to adverse outcomes, including calcium overload, activation of calpain-1, and cleavage of mitochondria-associated apoptosis-inducing factor. These processes ultimately exacerbated cardiac pathology and function in DSG2^mut/mut^ mice by promoting cellular damage and apoptosis [164]. In contrast, treadmill running (45 min, once daily, five days a week, for eight weeks) failed to reduce blood pressure, left ventricular hypertrophy, or myocardial fibrosis in aged spontaneously hypertensive rats (SHRs), showing no effect on mitochondrial dynamics. However, it effectively lowered blood pressure in younger SHRs [165]. Interestingly, swimming (45 min, twice daily, five days a week, for eight weeks) activated AKT, increased glycogen synthase kinase-3β phosphorylation, enhanced mitochondrial dynamics and spatial distribution, lowered ANP levels, and improved cardiac contractility [166].

**Figure 3.**
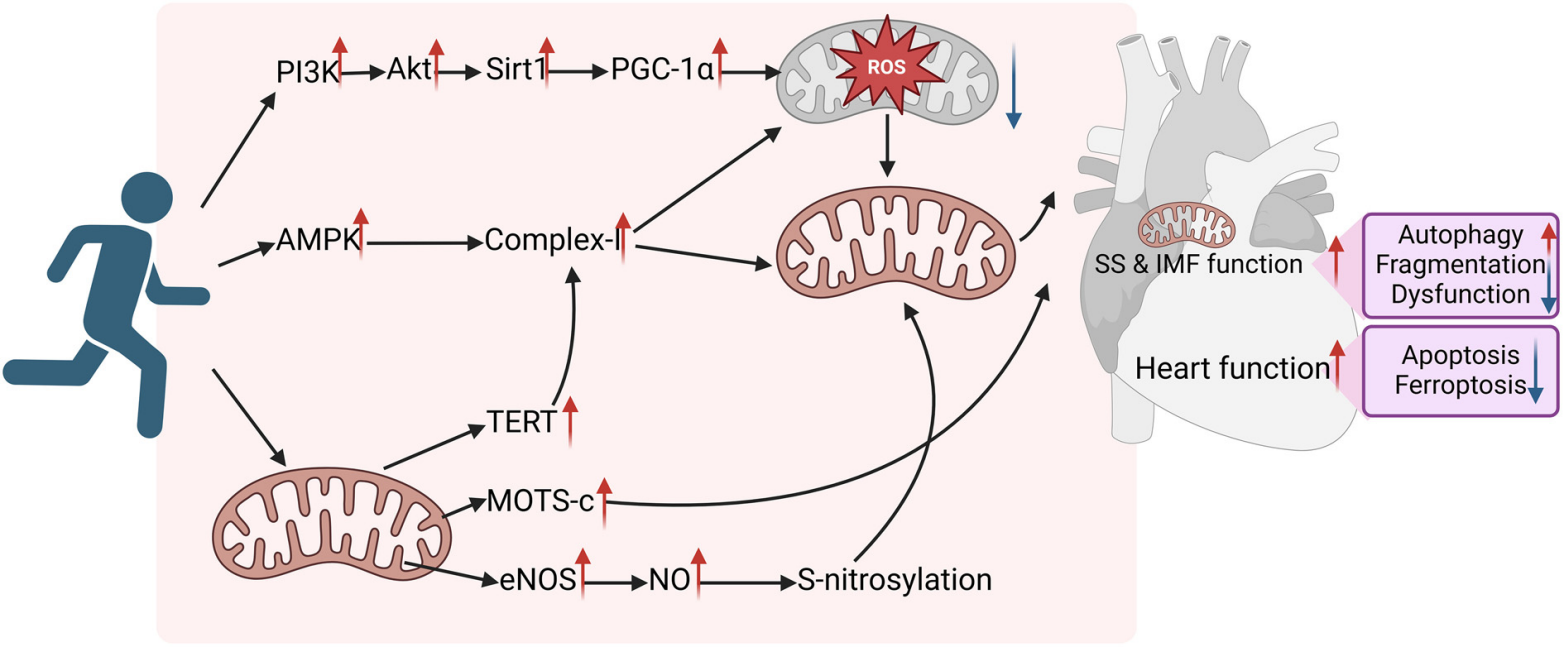
**Mechanisms through which exercise modulates mitochondria to enhance heart function.** Exercise can activate the SIRT1/PGC-1α/PI3K/Akt pathway and the AMPK/complex-I pathway in the body, as well as stimulate mitochondria to enhance the activity of MOTS-c, eNOS, and TERT. Consequently, these actions collectively promote overall mitochondrial function, increase mitochondrial autophagy, alleviate mitochondrial fragmentation and dysfunction, reduce cardiomyocyte apoptosis and ferroptosis, and ultimately improve cardiac function. PI3K: Phosphoinositide 3-kinase; AKT: Protein kinase B; Sirt1: Sirtuin 1; PGC-1α: Peroxisome proliferator-activated receptor gamma coactivator 1-α; Complex I: NADH dehydrogenase; TERT: Telomerase reverse transcriptase; MOTS-c: Mitochondrial-derived peptide; eNOS: Endothelial nitric oxide synthase; NO: Nitric oxide; SS: Subsarcolemmal mitochondrial populations; IMF: Intermyofibrillar mitochondrial populations.

These contrasting findings highlight the complexity of exercise’s cardiovascular effects, emphasizing the need for tailored exercise programs to optimize heart health in diverse populations (Figure 3).

### The influence of exercise on exerkines

Exerkines are fluid factors that respond to both acute and chronic exercise, playing a crucial role in delivering various cardiac metabolic benefits. Recently identified exerkines, such as FGF21, BAIBA, and irisin, offer new insights into how exercise provides both immediate and long-term cardiovascular advantages [167].

#### FGF21

FGF21 was initially identified as a liver-secreted protein, but later studies revealed that skeletal muscle, the pancreas, and brown adipose tissue can also produce it [168]. Research on FGF21−/− mice has shown that FGF21 overexpression can preserve myocardial mitochondrial dynamics and improve cardiac dysfunction caused by its deficiency [169]. While the heart was traditionally considered the primary target organ of FGF21, recent findings suggest that myocardial cell damage can trigger its autocrine release, providing cardioprotective effects [170]. Additionally, other tissues, such as the liver and brown adipose tissue, secrete FGF21 in a paracrine manner to inhibit ventricular remodeling and protect the heart [171, 172].

In mice with cardiomyocyte β-klotho knockout, endurance exercise led to an upregulation of FGF21, which activated AMPK, resulting in FOXO3 phosphorylation and increased SIRT3 expression. This cascade ultimately prevented mitochondrial dysfunction and improved cardiac function in diabetic cardiomyopathy [173].

Additionally, studies have shown that FGF21 has significant predictive value in distinguishing between mild and severe HF caused by T2DM. It can also serve as an independent predictor of late-stage HF in patients with HFrEF, HFpEF, HFmrEF, and T2DM [174–176]. At the same time, research on the therapeutic potential of FGF21 continues to advance. For instance, a study on insulin-resistant mice found that long-term FGF21 treatment activated the FAO signaling pathway, leading to improved cardiac metabolism, reduced insulin resistance, enhanced FGF21 sensitivity, and better overall cardiac function [177].

#### Irisin

Irisin, produced during skeletal muscle exercise and activated by PGC-1α, is derived from the cleavage of the membrane protein FNDC5 and serves as a key mediator of exercise-induced metabolic adaptations [178–180]. Recent studies have shown that Irisin can also be secreted by various tissues, including the heart, brain, liver, and fat, though its highest expression occurs in skeletal muscle. As a myokine, it plays a crucial role in enhancing energy expenditure and exerts its effects through both autocrine and paracrine mechanisms.

A study examining various exercise modalities—including endurance training, weight resistance, vibration, and electrical stimulation—found that all forms increased Irisin/FNDC5 levels in the myocardium, thereby promoting mitochondrial autophagy. Among these, weight resistance exercise had the most pronounced effect, significantly activating the PINK1/Parkin-LC3/P62 pathway, which in turn suppressed oxidative stress and improved cardiac function [181].

In models of radiation-induced heart injury, treadmill running (30-min sessions, once daily, five days a week, for three weeks) increased Irisin expression, selectively activated mitochondrial autophagy, and improved heart function [182]. This induced protective mitochondrial autophagy also alleviates cardiac hypertrophy caused by TAC and apoptosis triggered by Ang II [183, 184]. Iditarod, a protein related to FNDC5, is produced in response to exercise, modulating myocardial autophagy levels and enhancing resistance to exercise-induced cardiac stress [185]. In a type 2 diabetes mellitus model, treadmill training (45-min sessions, once daily, five days a week, for eight weeks) elevated Irisin levels, which in turn inhibited excessive mitochondrial fission mediated by DRP1 [186]. Research suggests that Irisin can serve as a biomarker for predicting the long-term clinical prognosis of HFpEF patients with low or near-normal NT-proBNP levels [187]. Additionally, it may help predict outcomes in chronic HF associated with T2DM, as well as in acute decompensated HF patients with acute myocardial infarction [188]. Furthermore, the precursor FNDC5 of Irisin shows increased expression in HF patients with better aerobic exercise performance, indicating a potential link between FNDC5 and exercise capacity in HF patients [189].

#### Other exerkines

BAIBA is a valine metabolite composed of L-BAIBA and D-BAIBA isomers. It is produced by skeletal muscles during exercise and reaches other organs through paracrine and autocrine signaling to exert its effects [190]. Studies indicate that BAIBA can improve diabetic cardiomyopathy, obesity-induced atrial fibrillation, and atrial remodeling by inhibiting oxidative stress, reducing inflammation, and increasing insulin sensitivity. Together, these mechanisms contribute to its cardioprotective effects [191, 192]. Endurance training (60-min sessions, once daily, five days a week, for eight weeks) has been shown to increase BAIBA levels. This elevation enhances miR-208b expression and AMPK phosphorylation, reducing myocardial apoptosis and mitochondrial dysfunction—ultimately improving heart function after myocardial infarction [193]. In human studies, higher circulating BAIBA concentrations have been negatively correlated with several cardiac metabolic risk factors, including elevated blood pressure, cholesterol levels, and insulin resistance [194].

CCDC80 is a signal peptide, and evidence supports its presence in various cell types [195–197]. However, its functions and secretion mechanisms remain complex, necessitating further studies to clarify the underlying processes [195]. Swimming (60-min sessions, once daily, five days a week, for 12 weeks) has been shown to stimulate the production of coiled-coil domain-containing protein 80 (CCDC80). This protein selectively inhibits the kinase activity of JAK2 and the STAT3 signaling pathway, thereby reducing Angiotensin II-induced cardiac fibrosis and hypertrophy in mice while preserving heart function [198]. Additionally, the study found that circulating CCDC80 levels increased following exercise in healthy individuals. However, it did not assess these levels in populations with pathological conditions such as HF, which would be crucial for understanding its broader role. Future research is needed to determine whether CCDC80 can predict individual responses to exercise in HF patients, particularly in relation to metabolic and cardiorespiratory health.

**Figure 4.**
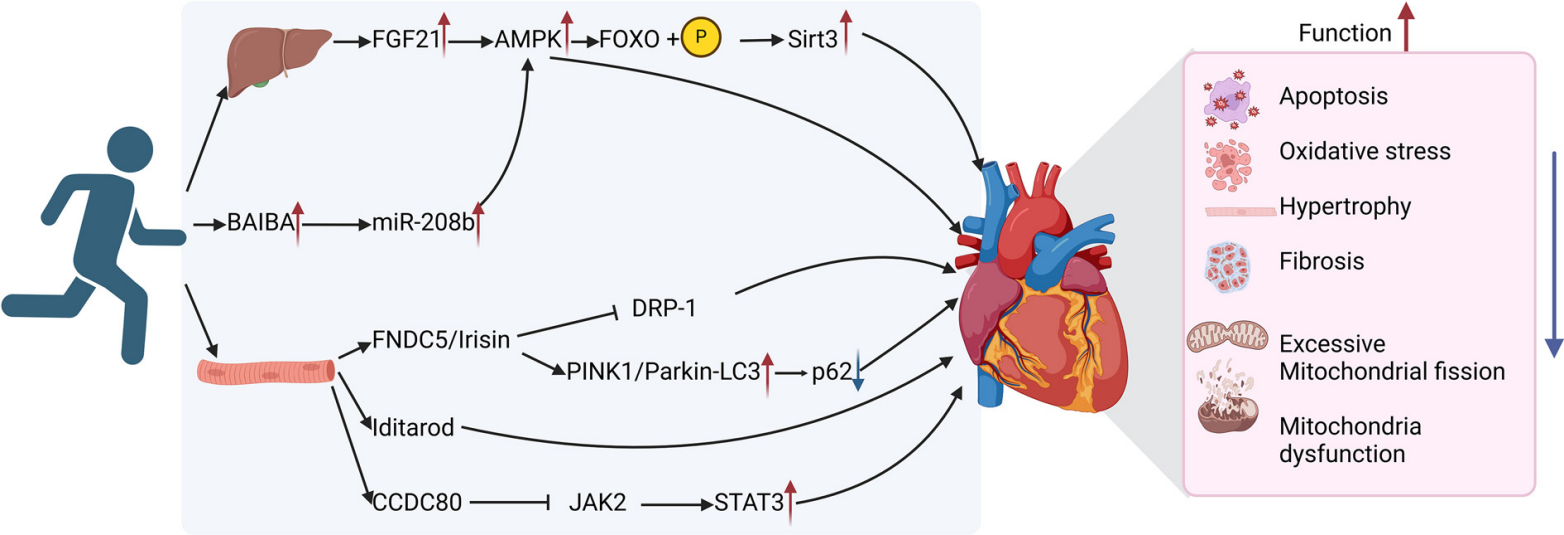
**Mechanisms of how exerkines enhance heart function.** Exercise can stimulate the body to secrete various exerkines, such as FGF21, BAIBA, Irisin, Iditarod, and CCDC80, which in turn collectively reduce adverse factors, such as cardiomyocyte apoptosis, oxidative stress, hypertrophy, fibrosis, excessive mitochondrial fission, and dysfunction, thereby enhancing cardiac function. FGF21: Fibroblast growth factor 21; AMPK: AMP-activated protein kinase; FOXO3: Forkhead box O3; Sirt3: Sirtuin 3; BAIBA: β-Aminoisobutyric acid; miR-208b: MicroRNA-208b; FNDC5: Fibronectin type III domain-containing protein 5; DRP1: Dynamin-related protein 1; PINK1: PTEN-induced kinase 1; CCDC80: Coiled-coil domain containing 80; JAK2: Janus kinase 2; STAT3: Signal transducer and activator of transcription 3.

In conclusion, exerkines, such as FGF21, Irisin, BAIBA, and CCDC80 contribute to cardiac protection and the recovery of heart function through various mechanisms (as illustrated in Figure 4). Given that HF patients often experience exercise intolerance, these exerkines hold significant potential as therapeutic alternatives to exercise. While research on their preclinical mechanisms continues to advance, direct clinical evidence for their use in HF patients is still lacking. Nevertheless, their potential remains promising.

## Conclusion

HF represents the final stage of multiple cardiovascular diseases and poses a significant global public health challenge, urgently requiring effective solutions. While numerous treatment strategies continue to be explored, a critical gap remains in identifying truly effective therapies. The cardiovascular benefits of exercise are well established, as it plays a key role in mitigating pathological factors, correcting myocardial metabolic imbalances, reducing oxidative stress and inflammation, minimizing cell death, and preserving mitochondrial function and dynamics. Maintaining normal heart function critically depends on optimal myocardial metabolism, as disruptions in energy supply or substrate availability can create an imbalance between myocardial energy demand and supply, ultimately leading to HF. Exercise not only increases myocardial metabolic demands but also dynamically reshapes the metabolic environment. Recent research highlights that exercise primarily exerts its protective effects by correcting metabolic disorders, enhancing glucose and lipid transport, preserving mitochondrial integrity, and promoting the release of beneficial exerkines.

Recent meta-analysis evidence indicates that exercise positively impacts quality of life (QoL), aerobic capacity, and cardiac function in elderly patients with HF. However, exercise variables, such as frequency, volume, and duration did not significantly improve the LVEF indicator [199]. This finding highlights the complexity of optimizing exercise programs for HF patients, as factors beyond exercise variables—or interactions between them—may play a crucial role in improving cardiac function. Additionally, the studies included in the meta-analysis exhibited considerable heterogeneity, likely due to differences in HF severity, exercise modalities, and the presence of comorbidities. This variability may obscure the dose-response relationship between exercise variables and LVEF improvement. Furthermore, research suggests that resistance exercise has a greater impact on enhancing aerobic capacity than aerobic exercise, while CT does not show a significant advantage over either approach in improving LVEF. These findings indicate that exercise prescriptions for HF patients should be personalized to maximize both safety and efficacy. At the same time, while improvements in LVEF following exercise interventions do not appear to be significantly related to exercise variables, it is essential to recognize that exercise-induced metabolic adaptations—such as enhanced mitochondrial function and reduced metabolic dysfunction—may still contribute meaningfully to overall cardiac health. Future research should integrate metabolomics and molecular analysis to better elucidate the mechanisms by which exercise benefits cardiac health, complementing traditional assessments such as echocardiography.

Current evidence indicates that moderate to low-intensity aerobic exercise benefits heart health, whereas exhaustive exercise can impair myocardial contractility, cause myocardial damage, and deteriorate heart function. This deterioration is closely linked to the type, intensity, and frequency of exercise [118, 134, 200, 201]. Therefore, defining exercise dosage limits is crucial. In clinical settings, exercise intolerance remains a major symptom in individuals with HF, significantly reducing their QoL. This intolerance likely stems from aging, reduced pulmonary reserve, and respiratory or skeletal muscle dysfunction, among other complex factors [202]. While current strategies to address exercise intolerance are limited, growing research suggests that exercise itself benefits HF and can help alleviate intolerance [203]. Thus, further studies on exercise dosage and timing are essential to optimize its role in cardiac rehabilitation, with important clinical and societal implications.

## Data Availability

Data sharing is not applicable to this article as no new data were created or analyzed in this study.
